# Successful concomitant minimally invasive surgery for aortic valve stenosis and right lung cancer via right mini-thoracotomy : A case report

**DOI:** 10.1186/s13019-022-01995-4

**Published:** 2022-10-03

**Authors:** Satoshi Sakakibara, Hiroyuki Nishi, Shinya Fukui, Mutsunori Kitahara, Kazuma Handa, Yumi Kakizawa, Takasumi Goto, Yasunobu Funakoshi

**Affiliations:** 1 Department of Cardiovascular Surgery, Osaka General Medical Center, 3-1-56 Bandai-higashi, Sumiyoshi-ku, 558-8558 Osaka, Japan; 2 Department of Thoracic Surgery, Osaka General Medical Center, Osaka, Japan

**Keywords:** Aortic valve replacement, Aortic valve stenosis, Lobectomy, Lung cancer, Concomitant minimally invasive surgery

## Abstract

**Background:**

The case of aortic valve stenosis complicated with lung cancer have compelled cardiovascular surgeons to make challenging. We report the first successful short-term outcomes of one-stage minimally invasive aortic valve replacement and video-assisted thoracoscopic surgery lobectomy through right mini-thoracotomy in a patient with synchronous bicuspid severe aortic valve stenosis which was unsuitable for transcatheter aortic valve implantation and right lung cancer.

**Case presentation:**

A 76-year-old man with severe aortic valve stenosis was diagnosed with lung cancer of the right upper lobe with stage IA2. Considering the potential risk of tumor metastasis, a one-stage surgical therapy for right lung cancer and type 0 bicuspid aortic valve stenosis was required; however, transcatheter aortic valve implantation was unsuitable due to a bicuspid aortic valve with severe calcification. Therefore, concomitant minimally invasive aortic valve replacement and lobectomy via right mini-thoracotomy were performed. The postoperative course was uneventful.

**Conclusion:**

Concomitant aortic valve replacement and right lobectomy via right mini-thoracotomy may reduce surgical invasiveness, leading to early recovery. This surgical strategy is a useful option, particularly for patients with aortic valve stenosis complicated with right lung cancer.

## Background

The incidence of concomitant cardiovascular diseases and lung cancer (LC) is increasing.[^1^] These cases have compelled cardiovascular surgeons to make challenging, individualized choices, integrating life expectancy and quality-of-life assumptions.^[[2]]^ Traditionally, such cases are surgically approached by staged or simultaneous open strategies that present significant morbidities and surgical risks.^[[3]]^ We report the first successful short-term outcomes of one-stage minimally invasive aortic valve replacement (MICS-AVR) and video-assisted thoracoscopic surgery (VATS) lobectomy through right mini-thoracotomy in a patient with synchronous bicuspid severe aortic valve stenosis (AS) and LC.

## Case report

**Fig. 1:**
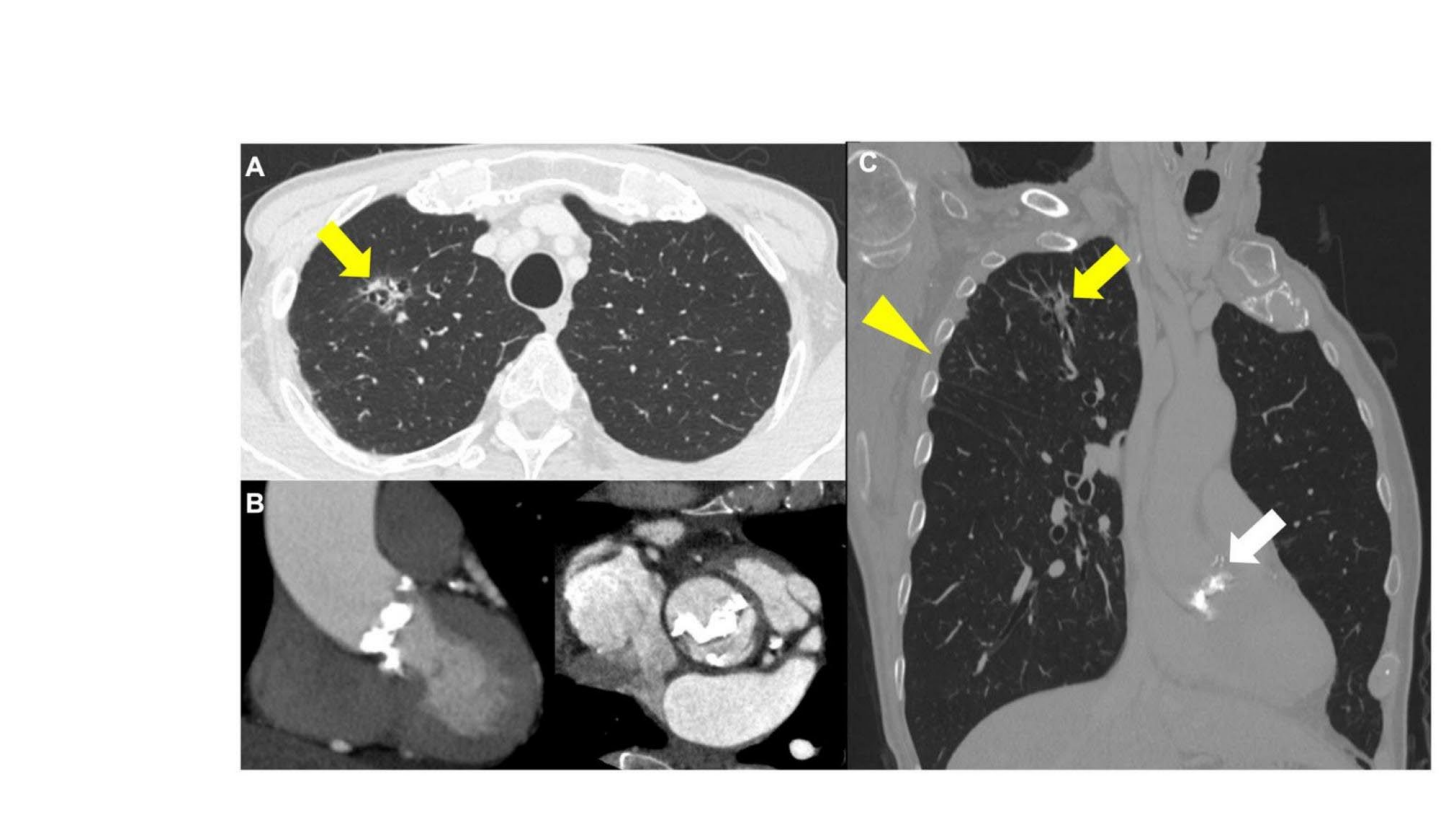
Preoperative CT findings. **A**: a nodular shadow in the right upper lobe without enlarged lymph nodes or distant metastases (yellow arrow). **B**: a severely calcified type 0 bicuspid aortic valve stenosis. **C**: the relationship lung cancer (yellow arrow), aortic valve(white arrow) and 4th intercostal space (yellow arrow head)

A 76-year-old man presenting with severe AS was admitted to our hospital for investigation of a nodular shadow which diameter was 13mm in the right upper lobe of the lung on computed tomography (CT) (Fig.1). Histopathological analysis by transbronchial biopsies demonstrated adenocarcinoma with stageIA2. Spirometry revealed %VC and FEV1.0% were 116.1% and 81.1%, respectively. Echocardiography showed a heavily calcified type 0 bicuspid aortic valve and severe AS with an orifice area, peak velocity, mean pressure gradient, left ventricular ejection fraction, stroke volume and stroke volume index of 0.59 cm^2^, 4.2m/s, 47 mmHg, 79%, 58ml and 39.2ml/m^2^ respectively. Given the decreased potential risk of tumor metastasis and early recovery to normal work, a one-stage procedure was desirable. However, transcatheter aortic valve implantation (TAVI) was unsuitable due to severe calcifications around the aortic root and annulus, which included a relatively high potential risk of aortic root rupture (Fig.1B). We considered that ND1b lymph node dissection would be enough because LC stage was relatively early. Therefore, we planned to perform concomitant MICS-AVR and right upper lobectomy through the same incision.

**Fig. 2:**
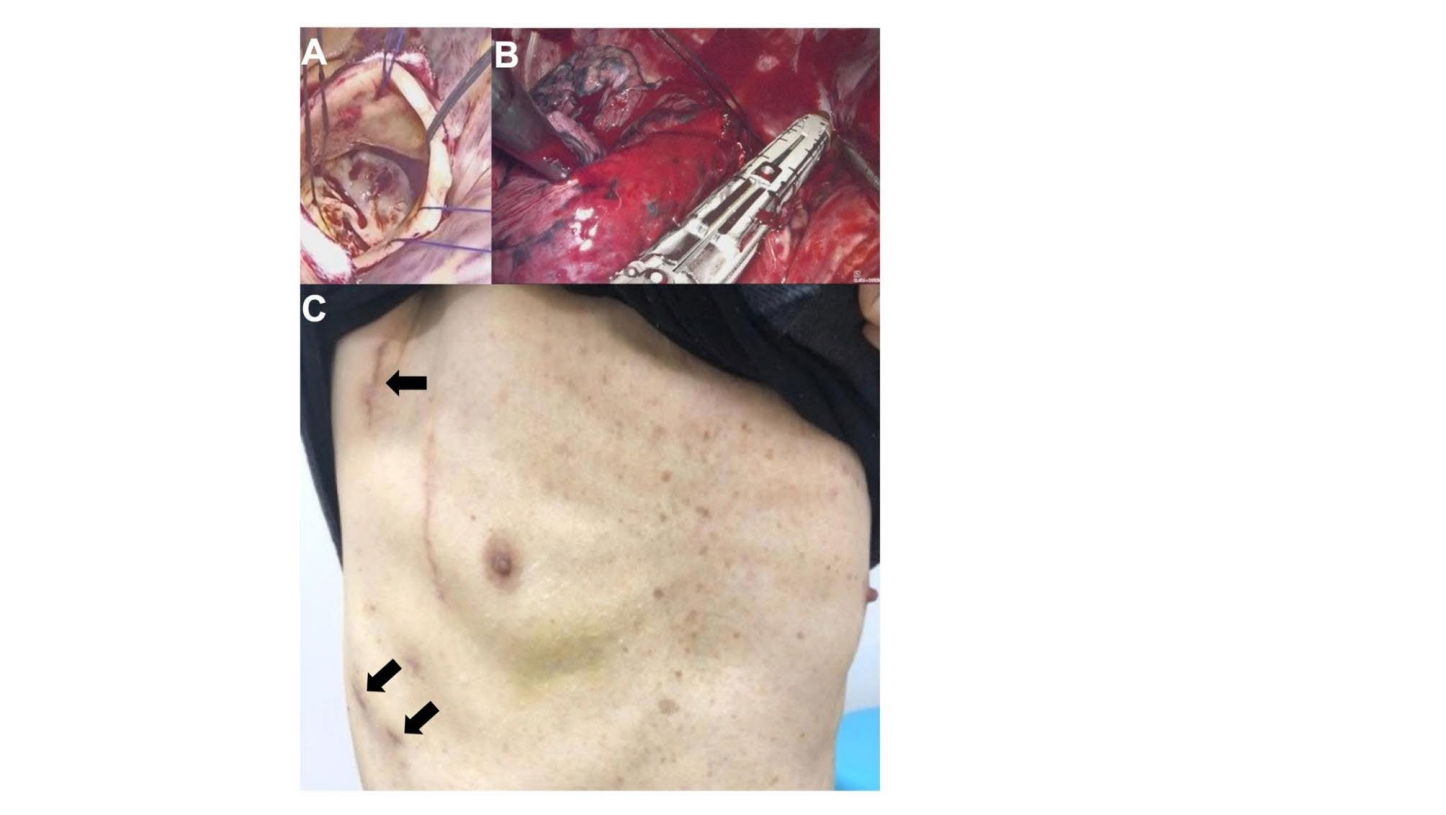
Intraoperative findings. **A**: Bicuspid aortic valve was with severe calcification (white arrow). The aortic valve was excised via right mini-thoracotomy. **B**: Right upper lobectomy was performed by VATS. **C**: Skin incisions; main thoracotomy was made at the lateral side on the edge of the pectoralis major muscle, and three right lateral mini-thoracotomies through the 4th and 7th intercostal spaces (black arrows)

After general anesthesia and double-lumen tube intubation, the patient was placed in the left decubitus position. This concomitant surgery was performed using usual instruments by which we use MICS-AVR and VATS procedure. A 7.5-cm skin incision was made at the lateral edge of the pectoralis major muscle along the anterior axillary line, and a right lateral mini-thoracotomy was performed through the fourth intercostal space. A videoscope was inserted through the port at the fourth intercostal space on the midaxillary line. Cardiopulmonary bypass (CPB) was established via the right femoral artery and vein (19- and 25-Fr Bio-Medicus NextGen Cannula, Medtronic Inc., MN, USA), and inserted left venting cannula via pulmonary vein. Cold blood cardioplegia was administered antegrade for the first time and selective for the second and subsequent times. Aortotomy was performed 2cm above the right coronary artery. The aortic valve was bicuspid, with severe calcifications (Fig.2A). After removal of the valve, AVR was performed using a 23-mm Mosaic Ultra aortic valve (Medtronic, MN, USA). This valve was implanted in the supra-annular position with 15 pledgeted mattress sutures. The patient was easily weaned from CPB, and protamine was systemically administrated.

Subsequently, with the insertion of two additional ports for VATS, a right upper lobectomy was performed using a GIA (Covidien Inc., MA, USA). The pulmonary vein and artery were cut. The upper lobe bronchus was divided using a GIA (Fig.2B). The right upper lobe and some lymph nodes (ND1b) were resected. The total operating time, CPB time, the time from cardiac arrest to return of heartbeat and VATS lobectomy time were 513, 193, 127 and 159min, respectively. The postoperative blood loss was 1020 ml and blood transfusion was 3370ml.

Postoperative histopathological analysis revealed adenocarcinoma with no lymph node metastases. The pathological stage was T1bN0M0 stage IA2. The postoperative course was uneventful. The intensive care unit stay and hospital stay were 1 and 12 days, respectively. He returned to normal work 24 days postoperatively.

## Discussion

The incidence of concomitant cardiovascular diseases and LC is increasing.^[[1]]^ The surgical strategy for each case needs to be considered based on the severity of AS, curability of LC, and patient safety. Lung surgery 4–6-week after AVR may lead to unreliability or metastasis. Conversely, lung surgery before AVR significantly increases the risks associated with anesthesia and death.^[[4]]^ Prior AVR for AS with lung cancer was often performed to avoid sudden hemodynamic change due to AS, but there was a report that showed prior lobectomy has been performed to avoid the risk of bleeding, even with protamine administration, and the risk of cellular immune dysfunction due to cardiopulmonary bypass which is associated with cancer progression.^[[5]]^ However, there is no significant evidence on the risk of cancer progression due to cardiopulmonary bypass, which is controversial. The concomitant procedure is not without limitations, such as the risk of dissemination due to the manipulation of the lung, inability to perform lymph node dissection, and excessive blood loss due to heparinization. We used the following points for the patient selection. Firstly, preoperative respiratory function is enough to tolerate to lung resection. Secondly, lung cancer is in the right lung. Finally, lung resection was limited to lobectomy or segmentectomy. A recent study revealed that TAVI for AS complicated with early cancer was effective and provided promising early results.^[[2]]^ Furthermore, some reports suggested that TAVI with simultaneous lung resection has good long-term results.^[[2, 6]]^ However, not every patient with AS can undergo TAVI due to a lack of anatomical suitability which includes severity and location of calcification etc. In this case, it was not appropriate owing to the anatomical morphology of the aortic valve. Considering the patient’s recovery and avoiding potential tumor dissemination, we decided to perform a one-stage minimally invasive surgery for AS and LC. We considered lobectomy before heparinization can increase the risk of pulmonary hemorrhage. In addition, taking into account the risk of hemodynamic collapse caused by AS during lobectomy, we performed AVR firstly. To the best of our knowledge, this is the first successful case of simultaneous MICS-AVR for severe AS and VATS oncologic resection via right mini-thoracotomy.

Conventional concomitant valve replacement and lung resection are usually performed with median sternotomy. However, lobectomy through median sternotomy is more difficult than the thoracotomy approach in terms of lymph node resection. If the surgery uses two different approaches, the patient will experience more postoperative discomfort. Conversely, minimally invasive valve surgery can be performed for valve diseases under CPB via a right mini-thoracotomy. Fujii et al. studied MICS-AVR under CPB via the femoral artery and femoral vein in Japan and reported no cases with postoperative neurological complications.^[[7]]^ Durdu et al. reported the efficacy of MICS-AVR with sutureless valve for bicuspid AS.^[[8]]^ We could not use sutureless valve in our institution, the sutureless valve can be an effective option. When the pulmonary nodule is located on the right lung, we can use the same incision on the right side to complete the lobectomy. Therefore, concomitant minimally invasive cardiac surgery and VATS lobectomy via right mini-thoracotomy are beneficial for and do not affect the safety of the operation while possessing cosmetic benefits. Furthermore, avoiding a sternum incision can reduce bleeding and the risk of wound infection, mediastinal infection,^[[9]]^ and sternal osteomyelitis.

## Conclusion

Although TAVI is a powerful option for patients with simultaneous AS and LC, the outcome of this patient who underwent one-stage MICS-AVR and VATS lobectomy was favorable. Given our case, particularly for patients with AS complicated with right LC who have unsuitable anatomical morphologies for TAVI, concomitant MICS-AVR and VATS lobectomy can be a safe and useful surgical option.

## Data Availability

available.

## Abberviations

AS: Aortic valve stenosis
LC: Lung cancer
MICS-AVR: Minimally invasive aortic valve replacement
VATS: Video-assisted thoracoscopic surgery
CT: Computed tomography
TAVI: Transcatheter aortic valve implantation
CPB: Cardiopulmonary bypass
